# Supplementary material for: Classification of breast cancer histopathological images using interleaved DenseNet with SENet (IDSNet)
Source: PLoS One. 2020 May 4;15(5):e0232127. doi: 10.1371/journal.pone.0232127 (PMC7198071; doi:10.1371/journal.pone.0232127)
Supplement: Attachment — Submitted filename: ResponseToReviewers.docx [file pone.0232127.s002.docx]

2020-0402, Stockholm

Re: PONE-D-20-01196
Classification of Breast Cancer Histopathological Images Using Interleaved DenseNet with SENet (IDSNet)
PLOS ONE

**Response to the Reviewers**

We are very grateful to the reviewer for the insightful comments on the paper. We have been able to incorporate changes to reflect the suggestions provided by reviewer. We have highlighted the changes in the manuscript with tracking. Here are the point-by-point response to the reviewer’s comments and concerns. For readability the reviewer’s original comments are included in bold italic font

Reviewer’s comment 1

***Fig.1 contains the classification sub-network, which contains pooling, BN and softmax function. Then, is the class probabilities obtained by the sub-network will be fed into a fully connected layer? This part is not clear. Please explain it.***

Revisions on lines 108-111:

To clarify the classification layer for the DensNet 121 architecture we added the following text “As described in the original DESNet paper [10], each conv layer corresponds to a composite sequence of operations consisting of batch normalization (BN)-Relu-Conv. The Classification sub-network includes 7x7 global average pooling, 1000D fully-connected layer, and softmax.”

Reviewer’s comment 2

***In experiments, the experimental setting is not clear, is the database are divided based on patients? In other words, is the training set and testing set containing the samples from one patient? Additionally, does the training set contain samples with different magnification factors when training one model?***

Revisions o lines 108-111:

To clarify the experimental setting, we added the following text “The division of the dataset was not subject or magnification specific. In other words, the images of different magnification factors from the different patients were mixed in the process of constructing the datasets for training, validating and testing”.

Reviewer’s comment 3

***Please seriously check the grammar errors and typos, some examples are:
1) state-of-art -> state-of-the-art
2) line 82: used also -> also used
3) line 182: very minute increase, please check this collocation.
4) line 323: Table II shows the experimental results for three previously… -> Table II shows experimental results of three previously …
5) line 334: varied -> is varied***

We corrected all spelling and grammatical errors pointed out by the reviewer. We have also carefully checked the grammar errors and typos. A number of additional revisions were made within the manuscript, which were highlighted in the marked document.
